# Analgesic effect of a mixed T-type channel inhibitor/CB_2_ receptor agonist

**DOI:** 10.1186/1744-8069-9-32

**Published:** 2013-07-01

**Authors:** Vinicius M Gadotti, Haitao You, Ravil R Petrov, N Daniel Berger, Philippe Diaz, Gerald W Zamponi

**Affiliations:** 1 Department of Physiology and Pharmacology, Hotchkiss Brain Institute, University of Calgary, Calgary, Canada; 2 Core Laboratory for Neuromolecular Production, The University of Montana, Missoula, MT, USA

**Keywords:** T-type Channels, Cannabinoid Receptors, Pain, Patch-Clamp, Mice

## Abstract

**Background:**

Cannabinoid receptors and T-type calcium channels are potential targets for treating pain. Here we report on the design, synthesis and analgesic properties of a new mixed cannabinoid/T-type channel ligand, NMP-181.

**Results:**

NMP-181 action on CB_1_ and CB_2_ receptors was characterized in radioligand binding and *in vitro* GTPγ[^35^S] functional assays, and block of transiently expressed human Cav3.2 T-type channels by NMP-181 was analyzed by patch clamp. The analgesic effects and *in vivo* mechanism of action of NMP-181 delivered spinally or systemically were analyzed in formalin and CFA mouse models of pain. NMP-181 inhibited peak Ca_V_3.2 currents with IC_50_ values in the low micromolar range and acted as a CB_2_ agonist. Inactivated state dependence further augmented the inhibitory action of NMP-181. NMP-181 produced a dose-dependent antinociceptive effect when administered either spinally or systemically in both phases of the formalin test. Both i.t. and i.p. treatment of mice with NMP-181 reversed the mechanical hyperalgesia induced by CFA injection. NMP-181 showed no antinocieptive effect in Ca_V_3.2 null mice. The antinociceptive effect of intrathecally delivered NMP-181 in the formalin test was reversed by i.t. treatment of mice with AM-630 (CB_2_ antagonist). In contrast, the NMP-181-induced antinociception was not affected by treatment of mice with AM-281 (CB_1_ antagonist).

**Conclusions:**

Our work shows that both T-type channels as well as CB_2_ receptors play a role in the antinociceptive action of NMP-181, and also provides a novel avenue for suppressing chronic pain through novel mixed T-type/cannabinoid receptor ligands.

## Background

In recent decades, our knowledge of the mechanisms underlying pain sensation has improved substantially; however, despite the increased understanding of the neurobiology of pain and the discovery of new pain-mediating molecules, only few novel classes of analgesic compounds have entered the clinic. Therefore, the identification of new pharmacophores for analgesics is of critical importance. Although the drug discovery sector frequently focuses on the design of highly specific channel and receptor modulators, the use of compounds that interact with more than one molecular target may provide opportunities for synergistic actions to increase analgesic efficacy [1].

Low-voltage activated (LVA) T-type calcium channels are essential contributors to signalling in electrically excitable cells [2-5] and are well recognized as important regulators of pain transmission [6-8]. T-type channels are highly expressed in primary afferent pain fibers with the highest expression levels in medium sized dorsal root ganglion (DRG) neurons [9]. Inhibition of T-type channels by intrathecal [7,10] or systemic [11] delivery of synthetic compounds, or through selective subunit knockdown *via* antisense oligonucleotides [7,12-14] has been shown to produce potent analgesic effects in rodents. Exactly how T-type channels contribute to pain processing is unclear, but may involve a regulation of the excitability of the primary afferent fiber and/or a contribution to neurotransmission at dorsal horn synapses [6,15,16]. Cannabinoid receptors on the other hand are G&nonBR;protein-coupled receptors [17] that are activated by cannabinoid ligands such as the phytocannabinoid Δ^9^-tetrahydrocannabinol (Δ^9^-THC) and endogenous cannabinoids such as anandamide and 2-arachidonyl glycerol (2-AG) [18]. These ligands bind to the two members of the CB receptor family - CB_1_ and CB_2_[19,20]. Cannanbinoids have shown efficacy in relieving pain in randomized-controlled trials often without serious adverse effects [21] and also they show therapeutic action in the treatment of pain associated with diseases such as multiple sclerosis [22,23]. Recent reports suggest that CB_1_ agonism can play a role in the analgesic effects of selective CB_2_ agonists in the rat CFA model [24]. A very low occupancy of CB_1_ receptors (<10%) by an agonist with a relatively low intrinsic efficacy can induce neurochemical and behavioral effects resulting in antinociception [25]. Remarkably, many endocannabinoids (such as anandamide) [26-28] and phytocannabinoids (Δ^9^-tetrahydrocannabinol and cannabidiol) [29,30] can also block T-type calcium channels, resulting in a more pronounced analgesia. This then suggests that such mixed cannabinoid receptor agonists with low intrinsic efficacy and T-type channel antagonists may produce synergistic actions with fewer side effects that may be exploited for analgesia.

**Figure 1:**
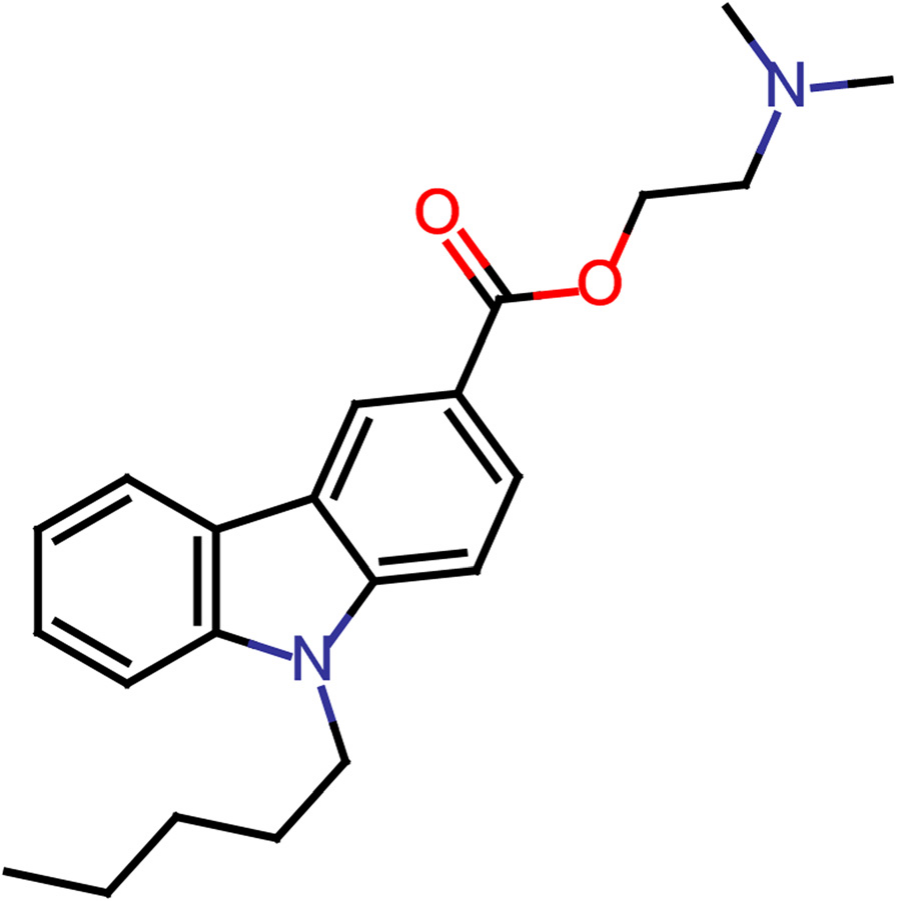
Molecular Structure of NMP-181.

In this study, we synthesized and pharmacologically characterized a novel compound NMP-181 (Figure 1) that exhibits a low intrinsic CB_2_ efficacy and potent T-type channel blocking activity. This compound was characterized in cell models, and was evaluated in various *in vivo* models for analgesic properties. Our data show that NMP-181 interferes with pain transmission through a mechanism related to CB_2_ receptor activation and Ca_V_3.2 channel inhibition but without nonspecific sedative actions, indicated by the inability of the active dose used in our pain model to affect the locomotor activity of mice on open-field test.

## Results

### In vitro characterization of putative tricyclic T-type channel inhibitors

**Figure 2:**
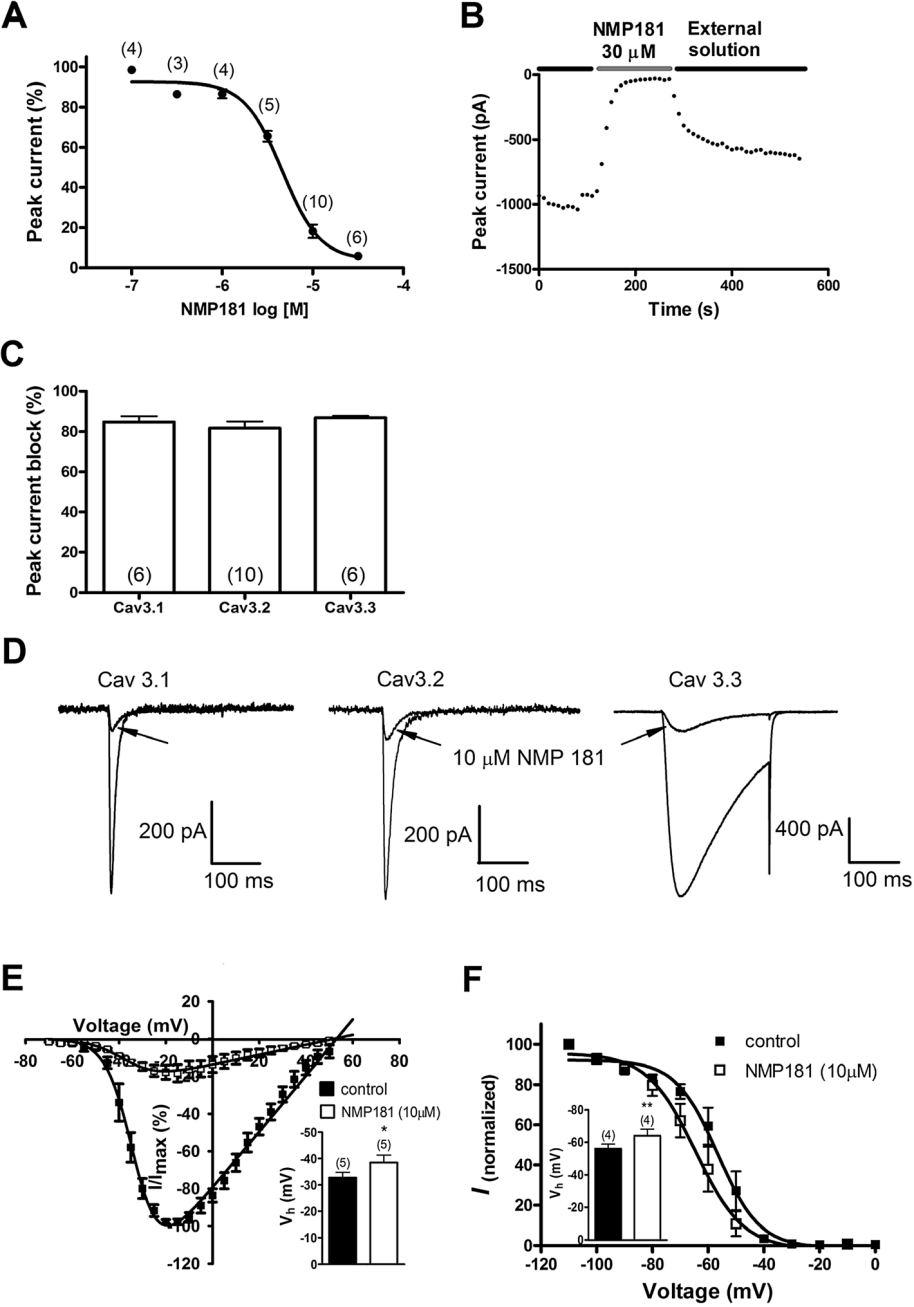
**Pharmacological and biophysical properties of NMP-181 block of T-type calcium channels. (A)** concentration dependence of NMP-181 inhibition of the Ca_V_3.2 peak current amplitude. The data were fitted with a Hill equation. The half-maximal inhibitory concentration (IC_50_) from the fit was 4.6 μM and the Hill coefficient was 2.1. Numbers in parentheses reflect numbers of experiments for each concentration point. **(B)** representative time course of the development of and recovery from NMP-181 (30 μM) inhibition. **(C)**, NMP-181 (10 μM) did not show selectivity on Ca_V_3 channels, as illustrated in the histogram and **(D)** representative traces from NMP-181 inhibition on Ca_V_3.1, 3.2 and 3.3 currents. Statistical significance was determined by one-way ANOVA followed by a Dunnett’s test, when the data were compared to those from Ca_V_3.2 group. **(E)** normalized current–voltage relations of Ca_V_3.2 current before and after application of 10 μM NMP-181. The half-activation potentials were -32.7 ± 2.0 mV and -38.4 ± 2.8 mV before and after application of NMP-181, respectively (inset, *P* < 0.05, paired *t* test). **(F)** steady-state inactivation curve obtained from Ca_V_3.2 channels before and after application of 10 μM NMP-181. The half-inactivation potentials were -56.0 ± 2.8 mV and -64.1 ± 4.0 mV before and after the treatment with NMP-181, respectively (inset, *P* < 0.01, paired *t* test).

We previously reported on a novel series of tricyclic compounds that were capable of interacting with both cannabinoid receptors and T-type calcium channels [31]. Based on our previous SAR data, we identified a core pharmacophore and synthesized NMP-181(Figure 1) as a possible dual CB_2_/T-type channel ligand. We first tested the ability of NMP-181 to inhibit transiently expressed T-type channels in tsA-201 cells. A concentration-response curve revealed that the inhibitory effect of NMP-181 on Ca_V_3.2 occurred with an IC_50_ of 4.6 μM and a Hill coefficient of 2.1, indicating cooperativity between multiple blocking modes (Figure 2A). Figure 2B illustrates the time-course of the effect of NMP-181 on Ca_V_3.2 peak current amplitude, revealing a rapid onset of block and only partial reversibility. To evaluate whether this compound was able to block other Ca_V_3 isoforms, 10 μM of NMP-181 was tested on transiently expressed human Ca_V_3.1 and Ca_V_3.3 channels at a test potential of -20 mV. As seen in Figure 2C,D, the degree of inhibition was similar for all three Ca_V_3 isoforms. Application of NMP-181 to Ca_V_3.2 channels produced a mild but significant hyperpolarizing in half-activation potential from -32.7 mV to -38.4 mV (n = 5, *P* < 0.05) (Figure 2E). Many of T-type channel blockers have state-dependent inhibitory effects, with enhanced potency at depolarized holding potentials [11,31,32]. To determine whether NMP-181 block is similarly state dependent, we recorded steady-state inactivation curves before and after application of NMP-181. As shown in Figure 2F, application of 10 μM of NMP-181 shifted the half-inactivation potential of Ca_V_3.2 channels towards more hyperpolarized potentials from -56.0 mV to -64.1 mV (n = 4, *P* < 0.01). These data imply that at a typical neuronal resting membrane potential additional T-type channel inhibition can occur due to a drug induced reduction in channel availability. Altogether, our data indicate that NMP-181 mediates T-type channel inhibition with affinities in the low micromolar range and possibly lower at more depolarized resting membrane potentials or as a result of frequency-dependent inhibition.

### Cannabinoid receptor affinity of NMP-181

NMP-181 was also tested for its cannabinoid activities using CB_1_ and CB_2_ binding assays. NMP-181 displaced respectively 65.4% and 60.5% of [^3^H]CP55,490 in HEK293 cells expressing human CB_2_ receptors, and in rat brain homogenates expressing CB_1_ receptors at 10 μM. NMP-181 exhibited the best affinity for CB_2_ receptors with a *K*_*i*_ value of 123 nM at human CB_2_ receptors and > 2 μM at rat CB_1_ receptors (Table 1**)**. NMP-181 exhibited low intrinsic activity at human CB_2_ receptors since only 24% of activation was detected at 1 μM in GTPγ[^35^S] functional assays. No functional activity was detected at lower concentrations. These data indicate that NMP-181 acts as a preferential agonist of CB_2_ receptors over the CB_1_ subtype.

**Table 1:** **Radioligand competitive binding assays and GTPγ[**^**35**^**S] functional activity**

**Ligand**	**Mean K**_**i**_		**GTPγ[**^**35**^**S] functional assays**			
	**Rat CB1(nM)**	**Human CB2 (nM)**	**Human CB1**		**Human CB2**	
			**EC**_**50 **_**(μM)**	**E**_**max **_**(%)**	**EC**_**50 **_**(μM)**	**% activation average at 1 μM**
NMP181	2238 ± 515	123 ± 28	NA	ND	>1	24%

*ND* not done, *NA* not active at 1 μM.

### Effect of NMP-181 on formalin-induced nociception

**Figure 3:**
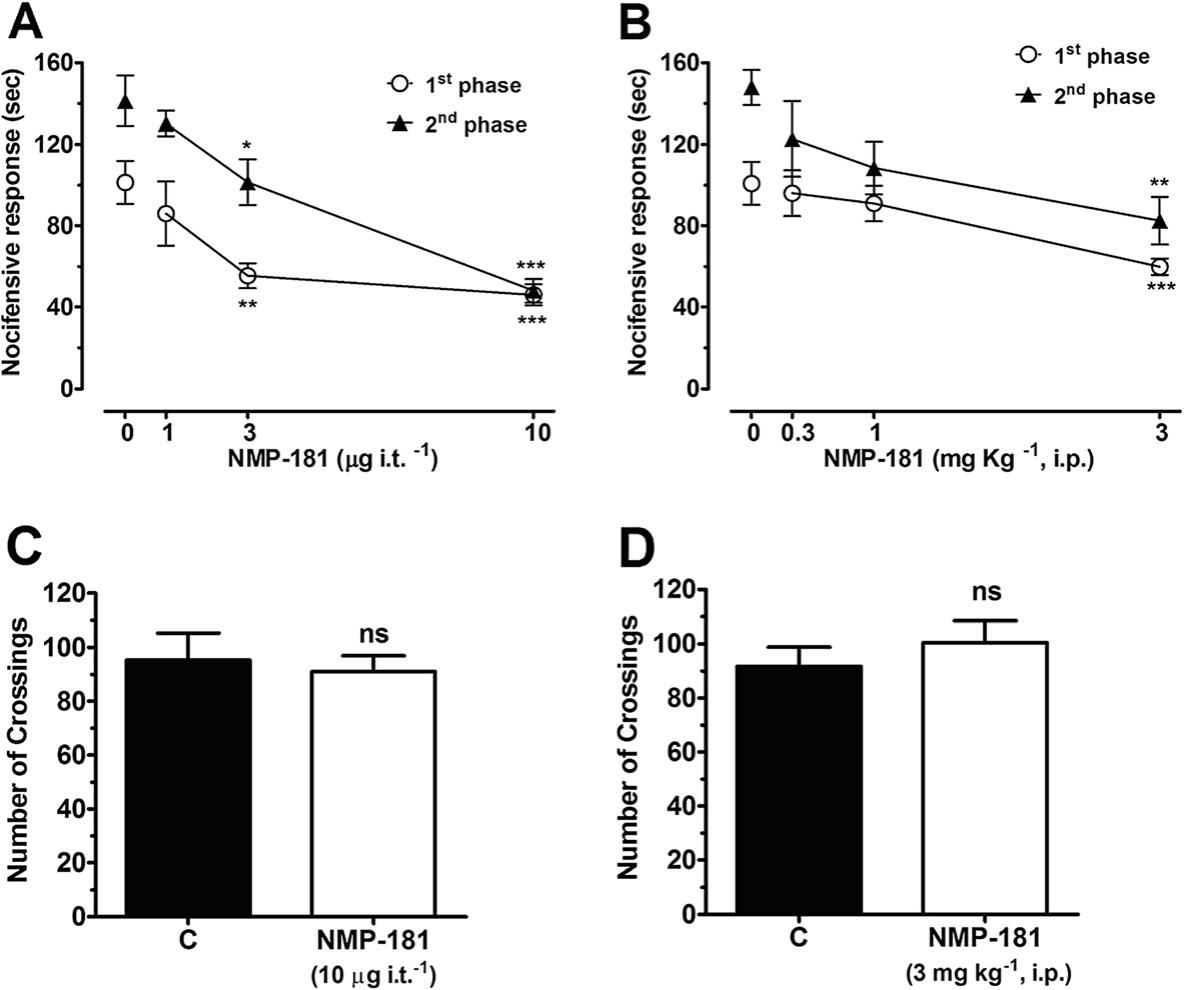
**Effect of NMP-181 administered by either i.t. (A,C) or i.p. (B,D) routes on the first and second phases of formalin-induced (A,B) pain and mice crossing in the open-field test (C,D).** Each bar or individual point represents the mean responses from 6–8 animals and the error bars indicate the S.E.M. Control values (indicated by “0”) are from animals injected with 5% of DMSO in PBS and the asterisks denote the significance relative to the respective control group. **P<0.01; ***P<0.001. (one-way ANOVA followed by Tukey’s test).

Given the agonist activity on CB_2_ receptors and the concomitant T-type channel antagonist activity, we hypothesized that this compound may show efficacy in models of inflammatory pain. NMP-181 was delivered by the i.t. route and its effects on both the acute nociceptive and the slower inflammatory pain phases of a formalin test were evaluated [33]. Mice were treated i.t. with NMP-181 or control solution (PBS + DMSO 5%) 15 minutes prior to behavioural assessment. One-way ANOVA revealed that i.t. treatment of mice with NMP-181 **(**1**–**10 μg i.t.^-1^) significantly reduced pain response time in both first (Figure 3A) and second (Figure 3B) phases (55±5% and 66±4% inhibition, respectively). Intraperitoneal (i.p.) treatment of mice 30 minutes before formalin injection resulted in a significantly (one-way ANOVA) decreased pain response time in both the first (Figure 3C) and second (Figure 3D) phases (41±3% and 44±8% inhibition, respectively). Importantly, neither spinal (*via* i.t.) nor systemic (*via* i.p.) treatment with NMP-181 affected locomotor activity of mice assessed *via* an open-field test (Figure 3E, F).

### Effect of NMP-181 on CFA-induced persistent inflammatory nociception

**Figure 4:**
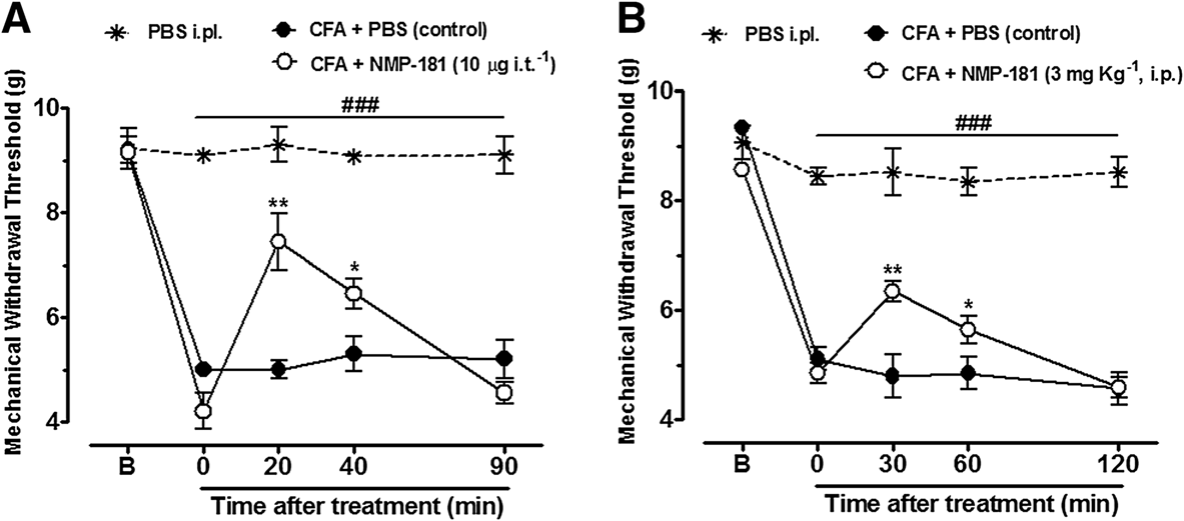
**Effect of NMP-181 delivered via i.t. (A) or i.p. (B) on the mechanical hyperalgesia induced by intraplantar delivery of CFA.** Each circle represents the mean responses from 7–8 animals and the error bars indicate the S.E.M. Control values (indicated by black circles) are from animals injected with 5% of DMSO in PBS and the asterisks denote the significance relative to the control group. *P<0.05; **P<0.01 and ^###^P<0.001 when compared to PBS intraplantar group (two-way ANOVA followed by Tukey’s test).

To ascertain whether NMP-181 modulates pain transmission under chronic inflammatory conditions, we analysed the nociceptive response of NMP-181 treated mice after CFA injection. As shown in Figure 4A, B, mice injected with CFA developed mechanical hyperalgesia 3 days after CFA injection as indicated by a significant decrease of withdrawal thresholds when compared to pre-CFA baseline levels of the control group (P < 0.001). Two-way ANOVA revealed that spinal treatment of mice with NMP-181 (10 μg i.t.^-1^) significantly attenuated the mechanical hyperalgesia induced by CFA when compared with the CFA + PBS (control) group, at 20 minutes (P < 0.01) and 40 minutes (P < 0.05) after NMP-181 treatment (Figure 4A). When mice were treated with NMP-181 systemically (1 mg kg^-1^, i.p.), mechanical hyperalgesia induced by CFA was significantly attenuated at 30 minutes (P < 0.01) and 60 minutes (P < 0.05) after treatment when compared with the CFA + PBS (control) group (Figure 4B). These data indicate that NMP-181 is a regulator of chronic inflammatory pain when delivered either through i.t or i.p. routes. Altogether, these data indicate that NMP-181 treatment specifically modulates pain signalling and mediates analgesia when delivered either spinally or systemically to mice.

### Analysis of mechanism of action of NMP-181

**Figure 5:**
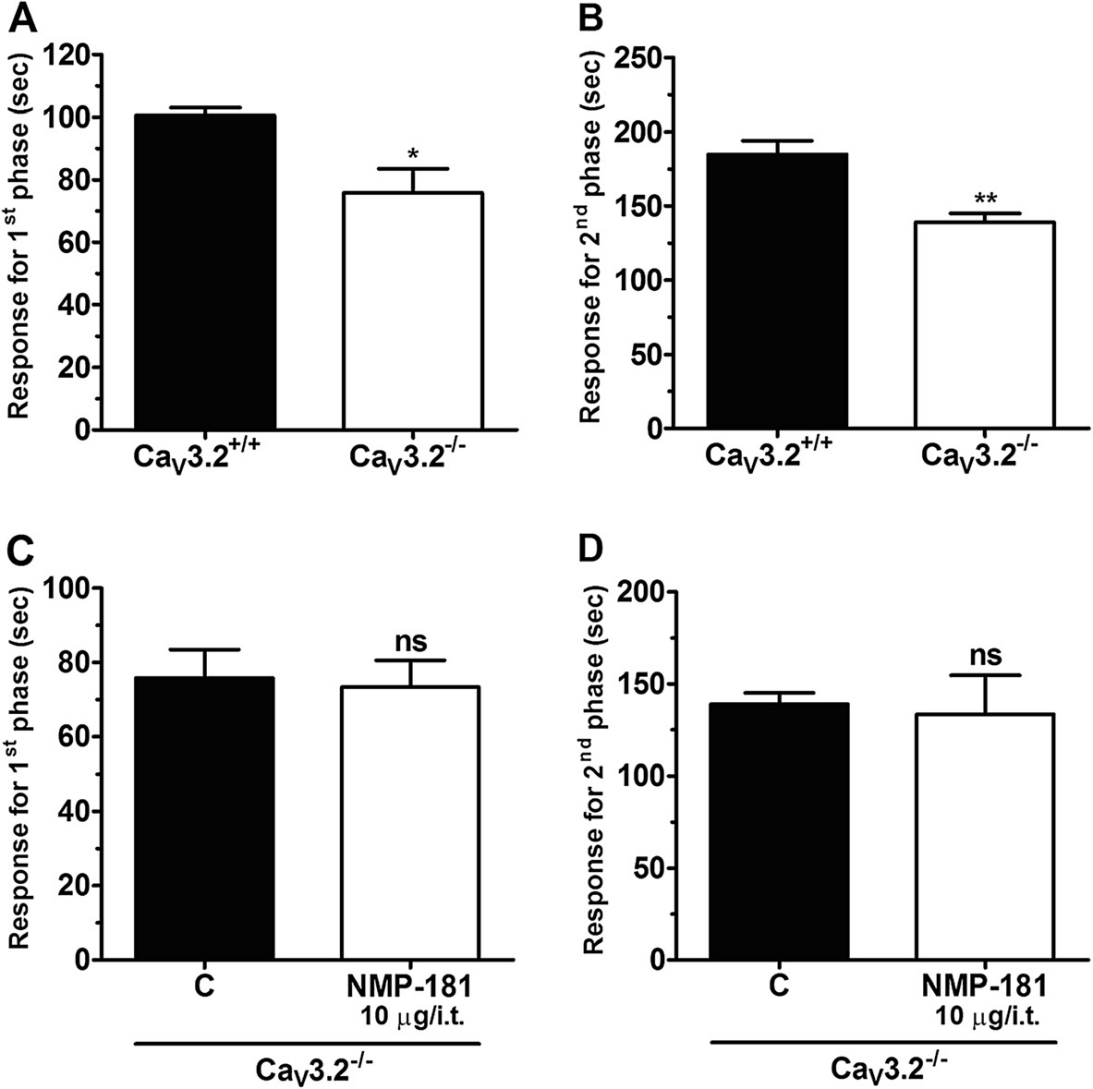
**Effect of NMP-181 delivered via i.t. to Ca**_**V**_**3.2 null mice on first (C) and second (D) phases of formalin induced pain.** Ca_V_3.2 null mice exhibit decreased pain response time when compared to wild-type animal on first **(A)** and second **(B)** phases of formalin induced pain. Each bar represents the mean responses from 6 animals and the error bars indicate the S.E.M. Control values (indicated by “C”) are from animals injected with 5% of DMSO in PBS (one-way ANOVA followed by Tukey’s test).

To investigate if T-type calcium channels play a role in the analgesic effect of NMP-181, we performed a formalin test in Ca_V_3.2 null mice that were treated either with vehicle or with NMP-181 (10 μg i.t.^-1^) and as shown in Figure 5A, B, Ca_V_3.2 null mice exhibited a lower mean response time when compared to wild-type mice, in agreement with previous data from [34]. As indicated in Figure 5C, D Ca_V_3.2 null mice appear to be completely insensitive to i.t. treatment with NMP-181 (10 μg i.t.^-1^) as revealed by one-way ANOVA. At face value, these data suggest that NMP-181 may predominantly inhibit pain signalling *via* T-type channel inhibition.

**Figure 6:**
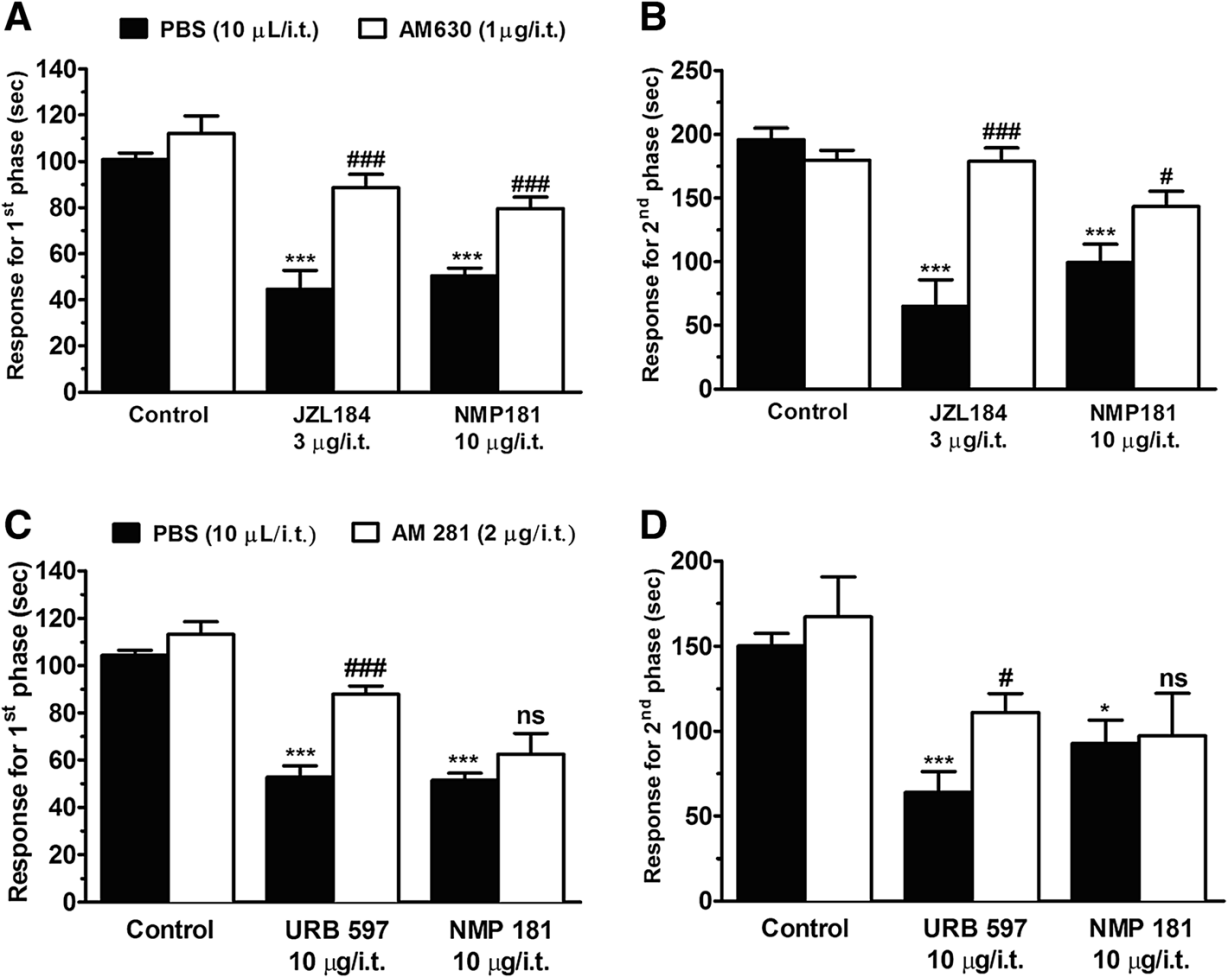
**Effect of i.t. treatment with selective CB**_**2 **_**(A, B) or CB**_**1 **_**(C, D) receptor antagonists on the antinociceptive action of NMP-181 on first (A, C) and second (B, D) phases of formalin-induced pain in mice.** Each bar represents the mean responses from 6–8 animals and the error bars indicate the S.E.M. Control values (indicated by “C”) are from animals injected with 5% of DMSO in PBS and the asterisks denote the significance relative to the control group. *P<0.05; ***P<0.001 and ^#^P<0.05; ^###^P<0.001 when compared to NMP-181 alone or positive controls treated groups (two-way ANOVA followed by Tukey’s test).

Intrathecal treatment of mice with the CB_2_ antagonist AM-630 (1 μg/i.t.), in combination with NMP-181 (10 μg/i.t.), significantly attenuated the antinociceptive action of NMP-181 (10 μg/i.t.) (Figure 6A, B) in both phases of formalin-induced pain as revealed by two-way ANOVA ([NMP-181 treatment F(1.65)=5.4, P <0.001 and NMP-181 × AM-630 + NMP-181 interaction: F(1.75)=4.5, P<0.001] for the first phase and [NMP-181 treatment F(2.87)=5.4, P <0.001 and NMP-181 × AM-630 + NMP-181 interaction F(1.67)=5.4, P<0.05] for the second phase). As a positive control, we used JZL-184 (3 μg/i.t., a dose which in itself produced a 50% reduction in response time; data not shown), an irreversible inhibitor for monoacylglycerol lipase, which is the primary enzyme responsible for degrading the endocannabinoid 2-arachidonoylglycerol. Although AM-630 is considered highly selective for CB_2_, it is remotely possible that AM-630 could somehow have affected NMP-181 block of T-type channels. However, the fact that NMP-181 was still active in Ca_V_3.2 KO mice supports the idea that NMP-181 acts via more than one target, and based on the AM-630 data, we thus conclude that CB_2_ receptors also contribute to the analgesic effect of NMP-181.

In contrast, intrathecal treatment of mice with the CB_1_ antagonist AM-281 (2 μg/i.t.), in combination with NMP-181 (10 μg/i.t.), did not modify the antinociception caused by NMP-181 (10 μg/i.t.) in the formalin test (Figure 6C, D), excluding an involvement of the CB_1_ receptor in the analgesic action of NMP-181. Importantly the dose of AM-281 used was able to significantly reverse the analgesic action of URB-597 (10 μg/i.t., a dose that resulted in a >50% reduction in response time, data not shown), an inhibitor of the enzyme fatty acid amide hydrolase, which is the primary degradatory enzyme for the endocannabinoid anandamide. Collectively these data show that intrathecal delivery of NMP-181 mediates its analgesic actions through a combination of Ca_V_3.2 channel inhibition and CB_2_ receptor stimulation.

## Discussion

After the identification of the CB_1_[19] and CB_2_[20] receptors for delta-nine-tetrahydrocannabinol (Δ^9^-THC) in mammals, various pharmaceutical strategies have attempted to explore the potential therapeutic properties of the cannabinoid system while minimizing its problematic side effects [35,36]. A significant problem surrounding the medical use of cannabis-related compounds is a concern regarding their CB_1_-mediated psychoactive effects and abuse potential [37]. The interest in developing compounds whose mechanism of action involves the CB_2_ receptors without CB_1_ involvement, and thus without CB_1_-mediated psychotropic side effects, still remains a goal in medical therapeutics. For this reason, selective CB_2_ receptor ligands appear as potentially viable compounds for pain management. CB_2_ activation suppresses microglial cell activation [38] and decreases neuroinflammation [39] which are common underlying mechanisms that lead to pathological pain [40] even though involvment of CB_1_ receptor cannot be excluded in these effects [24]. In addtion, cannabinoid receptors may couple to other effectors such as N-type calcium channels that are critical for the transmission of pain signals [41-43]. Interestingly, either Δ^9^-THC and cannabidiol [30] or the endogenous cannabinoid anandamide and its derivatives [26,28,44] inhibit T-type channel activity, thus mediating neuronal excitability *via* a receptor-independent mechanism. Given that blockade of the T-type channel subtype Ca_V_3.2 results in antinociceptive, anti-hyperalgesic, and anti-allodynic effects [11], the use of mixed CB_2_/T-type calcium channel ligand may provide a potential strategy for the development of more effective analgesics. Combining different mechanisms of action in a single drug could represent many advantages. These may include a gain of potency and duration of effects, a reduced number of prescribed drugs for a given condition, and perhaps a reduction of side-effects, as synergistic action on two targets may require an overall lower dose. We were thus interested in identifying a CB_2_ (but not CB_1_) agonist with T-type channel blocking ability. Here, we present a new compound of this class, NMP-181, which mediates analgesia *in vivo* dose-dependently and through a mechanism involving CB_2_ receptor activation and T-type calcium channel blockade.

Our results demonstrate that NMP-181 administered spinally (*via* i.t.) or systemically (i.p.) inhibits biphasic (neurogenic and inflammatory) pain induced by formalin in mice. This effect was greater during the second inflammatory pain phase of the test, indicating that NMP-181 strongly modulates inflammatory pain. In the formalin test, the neurogenic phase is elicited by direct activation of nociceptive terminals; whereas a combination of peripheral and central mechanisms underlies the inflammatory phase [33,45]. As chronic pain differs substantially from acute pain in terms of its persistence and adaptive changes such as neuroplasticity in the nervous system, we also assessed the action of NMP-181 in a persistent inflammatory pain model. Indeed, we demonstrated that NMP-181 delivered either spinally (*via* i.t.) or systemically (*via* i.p.) inhibited mechanical hyperalgesia induced by CFA at doses that did not seem to be directly associated with nonspecific sedative actions, indicated by the inability of these doses to affect the locomotor activity of mice in an open-field test. The CFA model of persistent pain produces central sensitization in response to the release of several pro-inflammatory mediators, which cumulatively increase the sensitivity of peripheral and central sensory pathways [46].

Our data reveal that the CB_2_ receptor, but not the CB_1_ receptor, is likely involved in the analgesic effects of NMP-181, as the effects of NMP-181 were partially reversed by AM-630, but not AM-281. At the same time, it is interesting to note that NMP-181 was completely ineffective in in Ca_V_3.2 null mice (Figure 5C, D), although the null mice themselves showed only a partial reduction in nocifensive behavior that in itself was smaller than the effect of NMP-181 in wild type animals (compare Figure 5A, B with Figure 3A, B). It is possible that Ca_V_3.2 null mice develop compensatory mechanisms that are insensitive to NMP-181. It is also possible that the NMP-181 mediated activation of CB_2_ receptors might trigger its analgesic effects in part *via* second messenger mediated inhibition of Ca_V_3.2 channel activity. This then might explain why null mice are completely insensitive to NMP-181 even though they presumably still express CB_2_ receptors. Finally, we note that the effects of NMP-181 on Ca_V_3.2 channels appeared to be state dependent, resulting in analgesia. Altogether, our data support a mechanism of which dual action of NMP-181 on CB_2_ receptors and Ca_V_3.2 calcium channels.

With regard to Ca_V_3.2, NMP-181 also mediated a hyperpolarzing shift in half inactivation potential that would be expected to produce additional inhibitory effects due to reduced channel availability at normal resting potentials. Such a feature is often associated with frequency dependent inhibition [6,32] which is a desirable feature in a drug designed to inhibit cellular excitability, such as in epilepsy [47], cardiac arrhythmias [48] and pain [30,49].

## Conclusions

Altogether, this study shows that NMP-181 exerts a rapid onset and pronounced antinociceptive effect in mice when administered spinally and systemically, at doses that do not interfere with locomotor activity. The NMP-181 scaffold may thus serve as a new pharmacophore for the development of new, more potent and longer-lasting mixed CB_2_/T-type channel ligands.

## Methods

### Chemical synthesis

NMP-181 was synthesized at the Core Laboratory for Neuromolecular Production. Full analytical data and detailed synthesis protocol are available in the supporting information (Additional file 1). NMP-181 was prepared in a four-step sequence starting with carbazole which was first alkylated and then formylated. Oxidation of the resulting aldhehyde followed by amidification afforded NMP-181. NMP-181 base was used for *in vitro* studies and NMP-181 hydrochloride was used for the *in vivo* studies.

### cDNA constructs

The cDNAs encoding human Ca_V_3.2 and Ca_V_3.3 were generously provided by Drs. Arnaud Monteil (CNRS Montpellier) and Terrance Snutch (University of British Columbia), respectively. The isolation of human Ca_V_3.1 cDNA in our laboratory was described previously [27]. The cDNA encoding the human CB_1_ receptor was isolated from a human brain stem cDNA library [50]. Sequencing confirmed that it was identical to GenBank Accession X54937. The coding sequence of the human CB_1_ receptor was subcloned as a HindIII-XbaI 1.5 kb DNA fragment in the expression vector pCDNA3 and in a bicistronic expression vector. The human CB_2_ receptor was cloned by PCR using oligonucleotides based on the sequence published by Munro and colleagues [20] with human genomic DNA as template. Sequencing of the resulting clones identified a fragment of 1.1 kb encoding the human cannabinoid 2 receptor, identical to GenBank Accession X74328. The coding sequence of the human CB_2_ receptor was inserted into bicistronic expression plasmids as a BamHI-NheI fragment and was subcloned as a BamHI-NheI DNA fragment in a BamHI-XbaI expression vector pCDNA3 (Invitrogen). The sequences of human CB_2_, used in the binding studies are the NCBI Reference Sequence.

### Cell culture and transfection

HEK293 cells and CHO cells were used in radioligand binding assays while tsA-201 cells were used in electrophysiological studies. Human CB_2_ (used in the binding studies) was cloned into pcDNA5.0FRT and cell lines were made using the FlpIn system from Invitrogen. tsA-201 cell culture and transient transfection of calcium channels were described previously [51]. In brief, Ca_V_3.1, 3.2 and 3.3 α1 subunits were transfected individually with yellow fluorescent protein in tsA-201 cells using the calcium phosphate method.

### In vitro receptor radioligand CB_1_ and CB_2_ binding studies

CB_1_ and CB_2_ radioligand binding data were obtained using National Institute of Mental Health (NIMH) Psychoactive Drug Screening Program (PDSP) resources as described earlier [31,52-54]. Compounds were screened in a competitive binding experiment using, respectively, membrane fractions prepared from rat brain homogenate expressing CB_1_ receptor and HEK293 cells expressing the human CB_2_ receptor. The competition binding experiment for CB_1_ and CB_2_ was performed in 96 well plates containing Standard Binding Buffer (50 mM Tris HCl, 1 mM EDTA, 3 mM MgCl_2_, 5 mg ml^-1^ fatty acid-free BSA, pH 7.4). The radioligand was [^3^H]CP55940, and the reference compound was CP55940. A solution of the compound to be tested was prepared as a 1 mg ml^-1^ stock in DMSO and then diluted in Standard Binding Buffer by serial dilution. Radioligand was diluted to five times the assay concentration in Standard Binding Buffer. Aliquots (50 μl) of radioligand were dispensed into the wells of a 96-well plate containing 100 μl of Standard Binding Buffer. Then, duplicate 50-μl aliquots of the test and reference compound dilutions were added. Finally, crude membrane fractions of cells were resuspended in 3 ml of chilled Standard Binding Buffer and homogenized by several passages through a 26 gauge needle, then 50 μl were dispensed into each well. The 250-μl reactions were incubated at room temperature for 1.5 hours, and then harvested by rapid filtration onto Whatman GF/B glass fiber filters pre-soaked with 0.3% polyethyleneimine using a 96-well Brandel harvester. Four rapid 500-μl washes were performed. Filters were placed in 6-ml scintillation tubes and allowed to dry overnight. Bound radioactivity was harvested onto 0.3% polyethyleneamine-treated, 96-well filter mats using a 96-well Filtermate harvester. The filter mats were dried, then scintillant was melted onto the filters and the radioactivity retained on the filters counted in a Microbeta scintillation counter. Raw data (dpm) representing total radioligand binding (i.e., specific + non-specific binding) were plotted as a function of the logarithm of the molar concentration of the competitor (i.e., test or reference compound). Non-linear regression of the normalized (i.e., percent radioligand resuspendedbinding compared to that observed in the absence of test or reference compound) raw data was performed in Prism 4.0 (GraphPad Software) using the built-in three parameter logistic model describing ligand competition binding to radioligand-labeled sites: y = bottom + [(top-bottom)/(1 + 10×-logIC_50_)] where the denominator equals the residual radioligand binding measured in the presence of 10 μM reference compound (i.e., non-specific binding) and the numerator equals the total radioligand binding observed in the absence of competitor. The log IC_50_ (i.e., the log of the ligand concentration that reduces radioligand binding by 50%) is thus estimated from the data and used to obtain the K_i_ by applying the Cheng-Prusoff approximation: K_i_ = IC_50_/(1 + [ligand]/K_D_) where [ligand] equals the assay radioligand concentration and K_D_ equals the affinity constant of the radioligand for the target receptor.

### GTPγ[^35^S] functional assays

Functional activity was evaluated using GTPγ[^35^S] assay in CHO cell membrane extracts expressing recombinant human CB_1_ or CB_2_ receptors as we previously described [31,55]. Compounds were solubilized in 100% DMSO at a concentration of 10 mM within 4 hours of the first testing session. A pre-dilution for the dose response curve was performed in 100% DMSO and then diluted 100 fold in assay buffer at a concentration 2 fold higher than the concentration to be tested. Compounds were tested for agonist activities in duplicate with CP55,940 (Tocris, Bioscience, Ellisville, MI, USA) as reference agonist. Membranes were mixed with GDP diluted in assay buffer to give 30 μM solution (volume:volume) and incubated for at least 15 min on ice. In parallel, GTPγ[^35^S] (GE Healthcare, Catalogue number SJ1308) were mixed with the beads (PVT-WGA (GE Healthcare, RPNQ001), diluted in assay buffer at 50 mg ml^-1^ (0.5 mg 10 μl^-1^) (volume:volume) just before starting the reaction. The following reagents were successively added in the wells of an Optiplate (Perkin Elmer): 50 μl of ligand, 20 μl of the membrane: GDP mix, 10 μl of assay buffer for agonist testing, and 20 μl of the GTPγ[^35^S]: beads mix. The plates were covered with a topseal, agitated on an orbital shaker for 2 min, and then incubated for 1 hour at room temperature. Then the plates were centrifuged for 10 min at 2000 rpm and counted for 1 min/well with a PerkinElmer TopCount reader. Assay reproducibility was monitored by the use of reference compound CP 55,940. For replicate determinations, the maximum variability tolerated in the test was of ± 20% around the average of the replicates. Efficacies (*E*_*max*_) for CB_1_ or CB_2_ are expressed as a percentage relative to the efficacy of CP 55,940.

### Electrophysiology

Methods and procedures used in the electrophysiological studies were described in detail by us previously [31]. Whole-cell currents were recorded from tsA-201 cells 2–4 days after transfection. NMP compounds were dissolved in DMSO at a 10 mM concentration and diluted into external recording solution with a final DMSO concentration no higher than 0.3%. Concentration-response studies were analyzed with the Hill equation *I*/*I*_*control*_ = 1/[1 + (*IC*_*50*_/[compound])^n^], where *I* is the normalized current at a given concentration of the compound, *IC*_*50*_ is the concentration of the compound yielding a current that is half of the control current, *I*_*control*_, and *n* is the Hill coefficient. For steady-state inactivation curves, data were fitted using Boltzmann equation *I* = 1/(1 + *e*^(*V*-*Vh*)/*k*^), where *V*_*h*_ is the half inactivation potential and *k* is the slope factor. Current–voltage (*I*-*V*) plots were fitted using the modified Boltzmann equation: *I* = 1/(1 + *e*^-(*V*-*Va*)/*k*^) × *G* × (*V* - *E*_*rev*_), where *E*_*rev*_ is the reversal potential, *G* is the maximum slope conductance, *k* is a slope factor, and *V*_*a*_ is the half activation potential.

### Animals

All experiments were conducted following the protocol approved by the Institutional Animal Care and Use Committee and all efforts were made to minimize animal suffering according to the policies and recommendations of the International Association for the Study of Pain. Adult male C57BL/6J (wild-type) or *CACNA1H* knockout (Ca_V_3.2 null) mice (20-25 g) were used (total of 265 mice). Animals were housed at a maximum of five per cage (30 × 20 × 15 cm) with *ad libitum* access to food and water. They were kept in controlled temperature of 23 ± 1°C on a 12 h light/dark cycle (lights on at 7:00 a.m.). When drugs were delivered by the intraperitoneal (i.p.) route, a constant volume of 10 ml/kg body weight was injected. When drugs were administered by intrathecal (i.t.) delivery, volumes of 10 μl were injected. Intrathecal (i.t) injections were given to fully conscious mice using the method previously described [56,57]. Animals were manually restrained, the dorsal fur of each mouse was shaved, the spinal column was arched, and a 30-gauge needle attached in a PE20 Polyethylene tube to a 25-μl Hamilton microsyringe (Hamilton, Birmingham, UK) was inserted into the subarachnoid space between the L_4_ and L_5_ vertebrae. Correct i.t. positioning of the needle tip was confirmed by a characteristic tail-flick response of animal. Appropriate vehicle-treated groups were also assessed simultaneously. All drugs were dissolved in DMSO and control animals received PBS + DMSO 5%, which was the maximum DMSO concentration in solutions delivered to animals. Mice with a targeted disruption of the Ca_V_3.2 gene (Homozygous *CACNA1H*, also called α_1_-3.2) [58] were purchased from Jackson Laboratories. Different cohorts of mice were used for each test and each mouse was used only once. The observer was blind to the experimental conditions in the experiment examining the action of NMP-181 on formalin, open-field and CFA tests (Figures 3 and 4).

### Formalin test

The formalin test allows us to evaluate two different types of pain: neurogenic pain (phase 1) is caused by direct activation of nociceptive nerve terminals, while inflammatory pain (phase 2) is mediated by a combination of peripheral input and spinal cord sensitization [33,45]. For this test, mice were acclimatized in the laboratory for at least 60 minutes before experiments. Animals received 20 μl of a formalin solution (1.25%) made up in PBS injected intraplantarily (i.pl.) in the ventral surface of the right hindpaw. Following i.pl. injections of formalin, the animals were immediately placed individually into observation chambers and the time spent licking or biting the injected paw was recorded and considered as a nociceptive response. We observed animals individually from 0–5 min (neurogenic phase) and 15–30 min (inflammatory phase) and the time spent licking or biting the injected paw was recorded with a chronometer.

### CFA-induced persistent inflammatory pain

In order to induce persistent inflammatory pain, mice received 20 μl of Complete Freund's Adjuvant (CFA) injected subcutaneously in the plantar surface of the right hindpaw (i.pl.) [59]. Control groups received 20 μL of PBS in the right paw. Animals received NMP-181 either spinally (1–10 μg i.t.^-1^) or systemically (0.3-3 mg kg^-1^, i.p.) 3 days following the CFA injection. Mechanical hyperalgesia was then measured using the Dynamic Plantar Aesthesiometer (DPA, Ugo Basile, Varese, Italy). Animals were placed individually in small enclosed testing arenas (20 cm × 18.5 cm × 13 cm, length × width × height) on top of a wire mesh floor. Mice were allowed to acclimate for a period of 90 minutes. The DPA device was positioned beneath the animal, so that the filament was directly under the plantar surface of the ipsilateral hind paw. Each paw was tested three times per session.

### Open-field test

The ambulatory behavior was assessed in an open-field test exactly as described previously [60]. The apparatus consisted of a wooden box measuring 40 × 60 × 50 cm with a frontal glass wall. The floor of the arena was divided into 12 equal squares and placed in a sound free room. Animals were placed in the rear left square and left to explore it freely for 6 min during which time the number of squares crossed with all paws (crossing) was counted. The apparatus was cleaned with a 10% alcohol solution and dried after each individual mouse session.

### Statistical analysis

For electrophysiological analyses, data values are presented as means ± SEM. Statistical significance was determined using paired *t*-tests for the comparison of channel biophysical properties before and after treatment. For behavioral analyses, each column represents the mean ± SEM and is representative of 2 independent experimental runs and evaluated by one-way, two-way or analysis of variance (ANOVA) followed by the appropriated Dunnett's or Tukey’s test. A value of P < 0.05 was considered to be significant.

## Abbreviations

Δ9-THC: Δ^9^-tetrahydrocannabinol; 2-AG: 2-arachidonyl glycerol; CB: Cannabinoid; CB1: Cannabinoid receptor type 1; CB2: Cannabinoid receptor type 2; CFA: Complete Freund's adjuvant; DPA: Dynamic plantar aesthesiometer; DRG: Dorsal root ganglion; LVA: Low-voltage activated.

## Competing interests

The authors declare that they have no competing interests.

## Author contributions

VMG, HY, RRP and NDB performed experiments and analyzed data. VMG, PD and GWZ designed experiments. VMG, PD and GWZ wrote the manuscript. The authors read and approved the final manuscript.

## Supplementary Material

Additional file 1 Details on synthesis of compounds. Click here for file
