# Supplementary material for: Analgesic effect of a mixed T-type channel inhibitor/CB2 receptor agonist
Source: Mol Pain. 2013 Jul 1;9:32. doi: 10.1186/1744-8069-9-32 (PMC3703287; doi:10.1186/1744-8069-9-32)

Analgesic effect of a mixed T-type channel inhibitor/CB_2 receptor agonist

Vinicius M. Gadotti[1], Haitao You[1], Ravil R. Petrov[2], Nelson D. Berger[1], Philippe Diaz[2] and Gerald W. Zamponi[1,*]

[1]Department of Physiology and Pharmacology, Hotchkiss Brain Institute,

University of Calgary, Calgary, Canada

[2]Core Laboratory for Neuromolecular Production, The University of Montana, Missoula, MT, USA

[*]Alberta Innovates Health Solutions Scientist and a Canada Research Chair in Molecular Neurobiology. To whom correspondence should be addressed. Tel.: 403-220-8687; Fax: 403-210-8106; E-mail: zamponi@ucalgary.ca.

Synthesis of 2-(Dimethylamino)ethyl 9-pentyl-9*H*-carbazole-3-carboxylate hydrochloride (NMP181). iii

^{1}H NMR of NMP181 hydrochloride in DMSO-d_6, Bruker 400 v

^{13}C NMR of NMP181 hydrochloride in DMSO-d_6, Bruker 400 vi

^{13}C DEPT135 NMR of NMP181 hydrochloride in DMSO-d_6, Bruker 400 vii

^{13}C DEPT90 NMR of NMP181 hydrochloride in DMSO-d_6, Bruker 400 viii

NMP181 (5.88 min, 100%, ESI: *m/z* 353.2029 (M + H)$^+$) ix

Synthesis of 2-(Dimethylamino)ethyl 9-pentyl-9*H*-carbazole-3-carboxylate hydrochloride (NMP181).

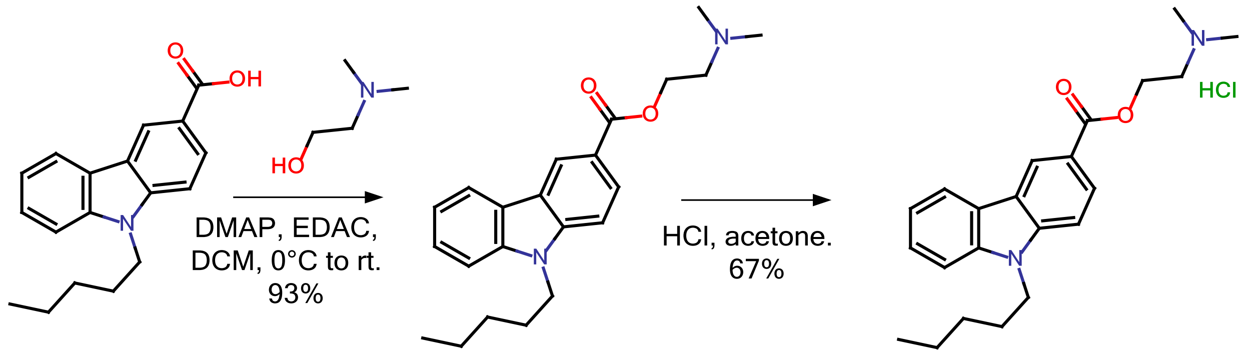

9-Pentyl-9*H*-carbazole-3-carboxylic acid (**1**) (100 mg, 0.36 mmol), 2-(dimethylamino)ethanol (36 µL, 0.36 mmol), and DMAP (87 mg, 0.71 mmol) were added to DCM (20 mL) under nitrogen. EDC (200 mg, 1.04 mmol) was added to the solution, and the reaction mixture was stirred for 16 h while warming at room temperature. The solvent was removed *in vacuo*, and the obtained residue was extracted with EtOAc (100 mL). The organic layer was washed consecutively with concentrated sodium bicarbonate (50 mL × 3), brine (50 mL), dried over magnesium sulfate, filtered, and concentrated *in vacuo*. The residue was purified on a Biotage® KP-NH cartridge (amino-modified silica gel) using heptanes/EtOAc in different proportions to afford the title compound as a clear, light yellow viscous oil (117 mg, 93%). ^{1}H NMR (500 MHz, $CDCl_3$) δ 8.81 (d, *J* = 1.5 Hz, 1H), 8.16 (dd, *J* = 8.6, 1.7 Hz, 1H), 8.12 (d, *J* = 7.7 Hz, 1H), 7.49 – 7.43 (m, 1H), 7.36 (d, *J* = 8.2 Hz, 1H), 7.33 (d, *J* = 8.6 Hz, 1H), 7.28 – 7.23 (m, 1H), 4.48 (t, *J* = 5.9 Hz, 2H), 4.21 (t, *J* = 7.2 Hz, 2H), 2.76 (t, *J* = 5.9 Hz, 2H), 2.36 (s, 6H), 1.81 (p, *J* = 7.3 Hz, 2H), 1.34 – 1.26 (m, 4H), 0.84 (t, *J* = 6.9 Hz, 3H). ^{13}C NMR and DEPT (126 MHz, $CDCl_3$) δ 167.46 (C=O), 143.17 (C), 141.03 (C), 127.38 (CH), 126.35 (CH), 123.00 (CH), 122.58 (C), 120.71 (CH), 120.68 (C), 119.84 (CH), 109.17 (CH), 108.19 (CH), 62.89 (CH_2), 58.14 (CH_2), 46.04 (CH_3), 43.25 (CH_2), 29.39 (CH_2), 28.67 (CH_2), 22.51 (CH_2), 14.02 (CH_3). ESI: *m/z* 353.1 $(M + H)^+$. HRMS calcd for $C_{22}H_{29}N_2O_2$ $(M + H)^+$ 353.2229, found 353.2260.

Compound **NMP181** was also isolated as a hydrochloride salt by adding aqueous hydrochloric acid (1 equiv) to a vigorously stirred solution of NMP181 in acetone at 0 °C. After 1 h the

solvent was removed by reduced pressure, and the obtained residue was sonicated in anhydrous diethyl ether for 30 min. The resulting precipitate was collected by filtration, washed with diethyl ether, and dried overnight in high *vacuo* to produce the title compound as a white solid (67%); mp. 133-136 °C.

^{1}H NMR of NMP181 hydrochloride in DMSO-d_6, Bruker 400

Parameter	Value
Solvent	DMSO
Pulse Sequence	zg30
Acquisition Date	2012-12-11T00:34:47
Temperature	296.1
Number of Scans	16
Spectrometer Frequency	400.18
Spectral Width	4835.6
Lowest Frequency	-44.7
Nucleus	1H
Acquired Size	32768
Spectral Size	65536

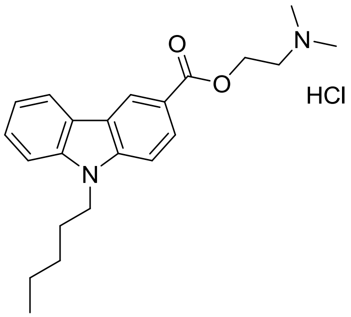

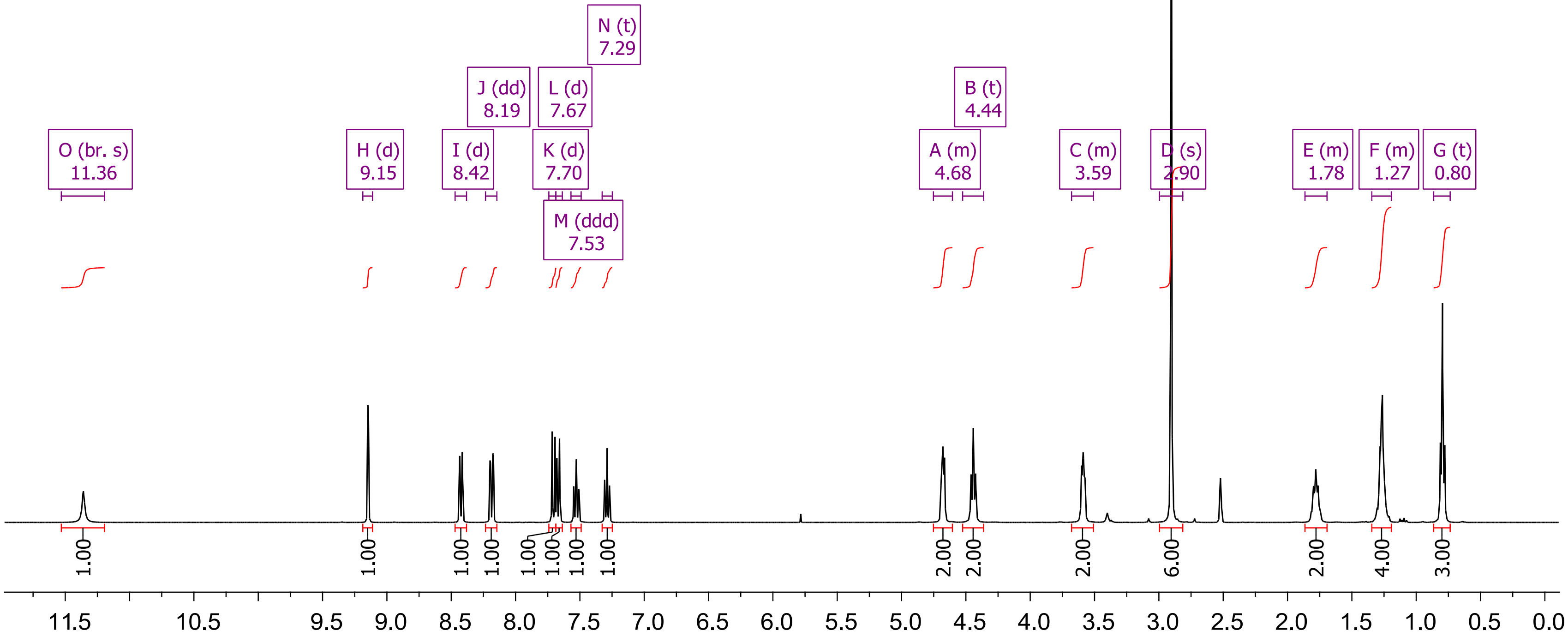

^{13}C NMR of NMP181 hydrochloride in DMSO-d_6, Bruker 400

Parameter	Value
Solvent	DMSO
Pulse Sequence	zgpg30
Acquisition Date	2012-12-11T01:10:14
Temperature	296.8
Number of Scans	512
Spectrometer Frequency	100.63
Spectral Width	17241.4
Lowest Frequency	-166.7
Nucleus	13C
Acquired Size	32768
Spectral Size	65536

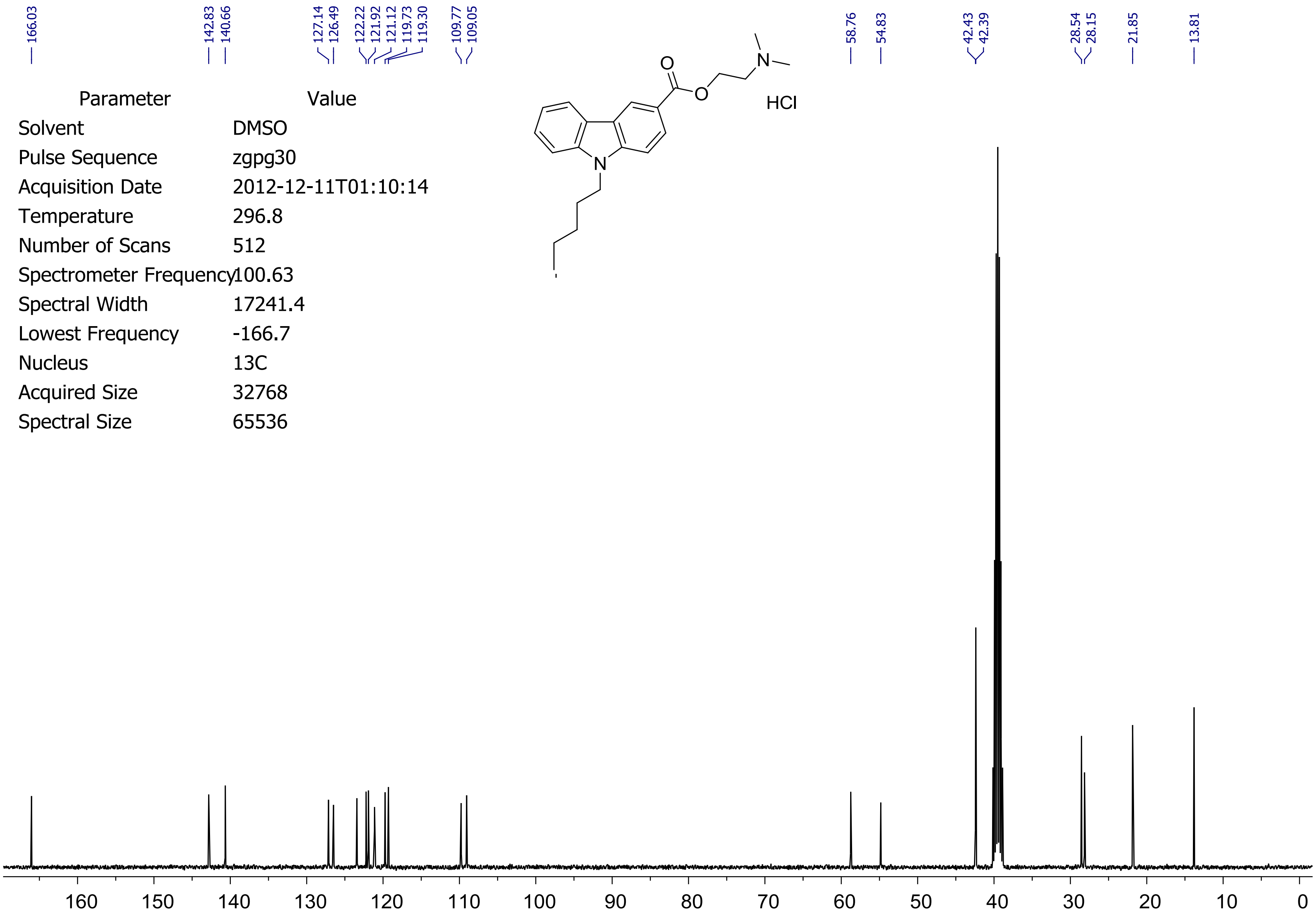

^{13}C DEPT135 NMR of NMP181 hydrochloride in DMSO-d_6, Bruker 400

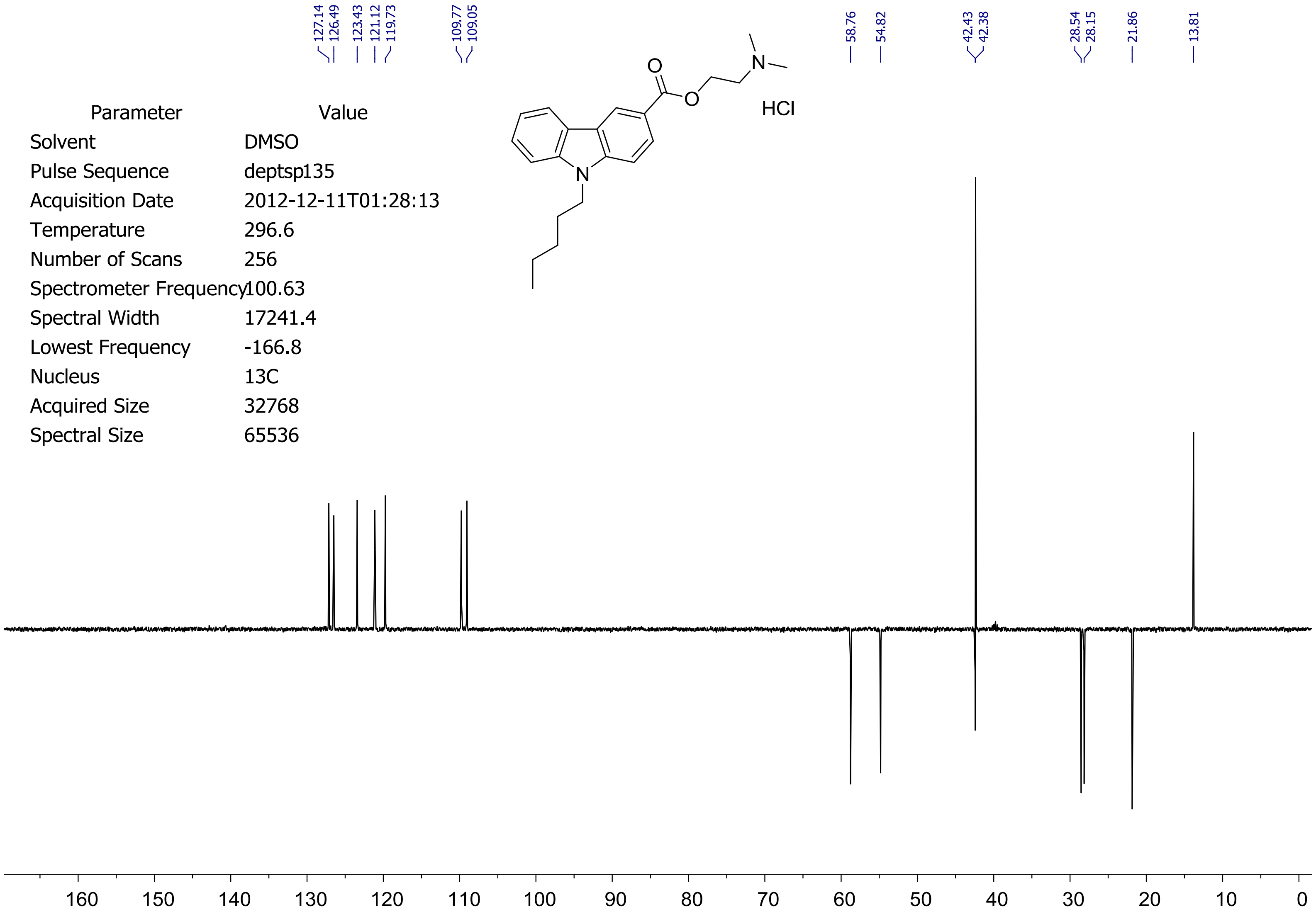

Parameter	Value
Solvent	DMSO
Pulse Sequence	deptsp135
Acquisition Date	2012-12-11T01:28:13
Temperature	296.6
Number of Scans	256
Spectrometer Frequency	100.63
Spectral Width	17241.4
Lowest Frequency	-166.8
Nucleus	13C
Acquired Size	32768
Spectral Size	65536

^{13}C DEPT90 NMR of NMP181 hydrochloride in DMSO-d_6, Bruker 400

Parameter	Value
Solvent	DMSO
Pulse Sequence	dept90
Acquisition Date	2012-12-11T01:46:12
Temperature	296.5
Number of Scans	256
Spectrometer Frequency	100.63
Spectral Width	17241.4
Lowest Frequency	-167.1
Nucleus	13C
Acquired Size	32768
Spectral Size	65536

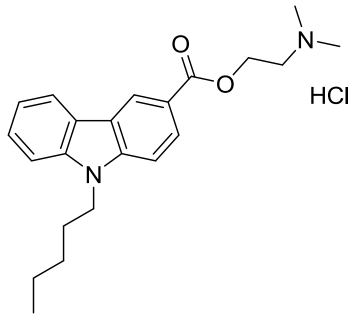

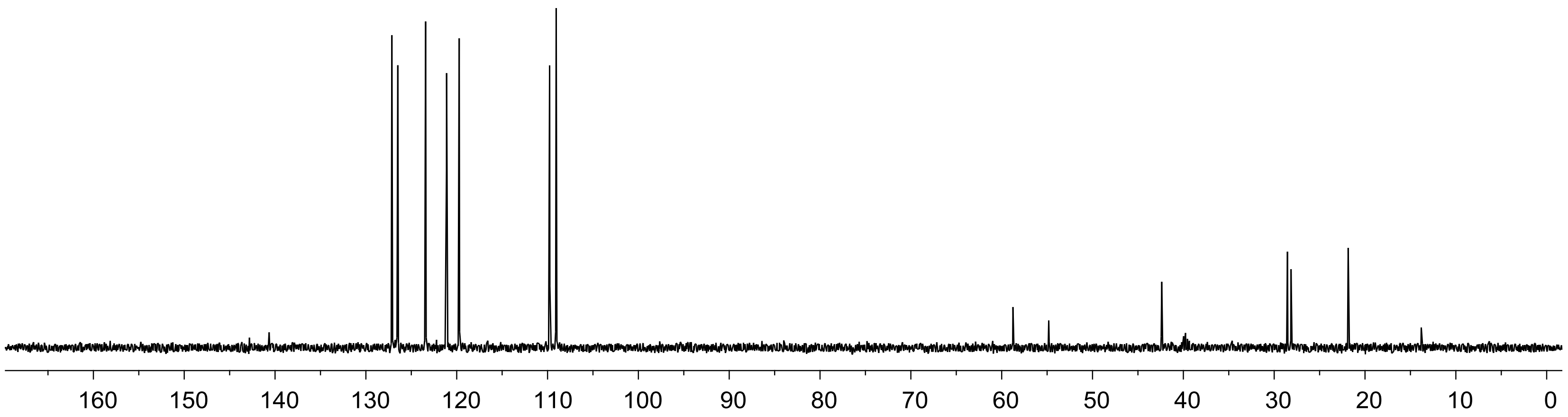

NMP181 (5.88 min, 100%, ESI: *m/z* 353.2029 (M + H)$^+$)

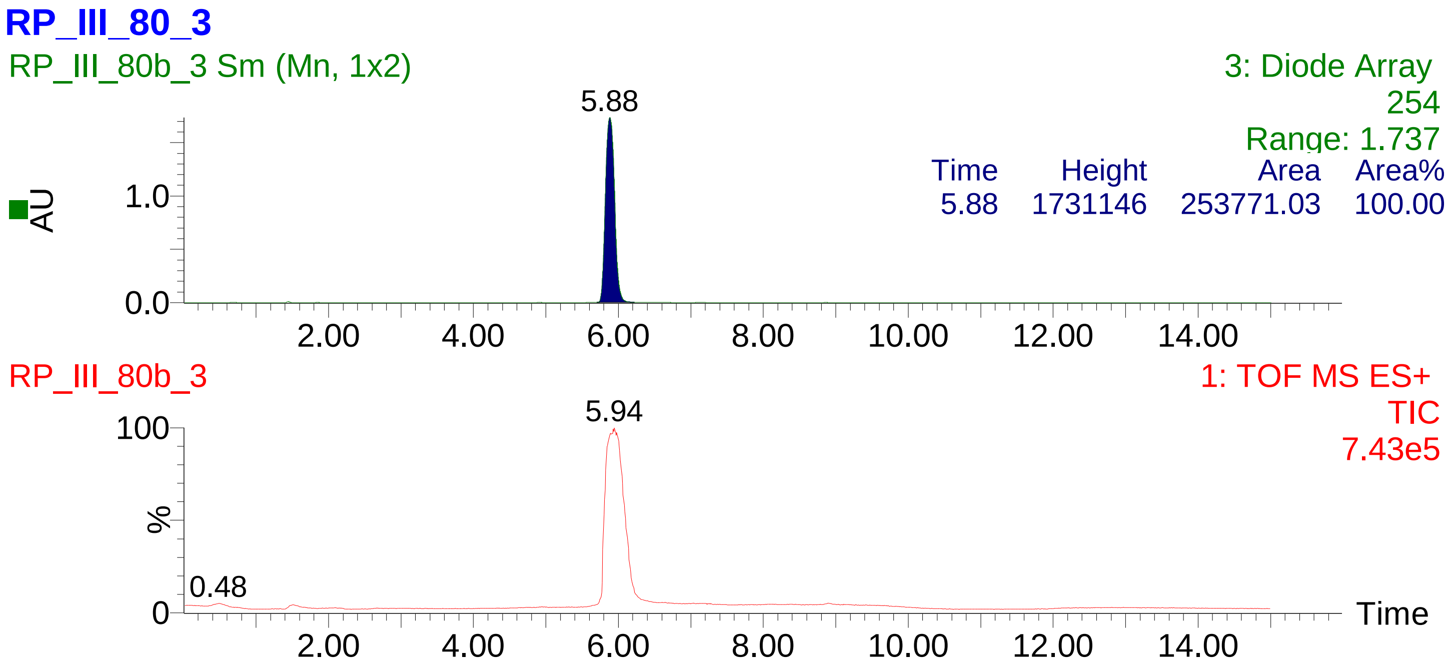

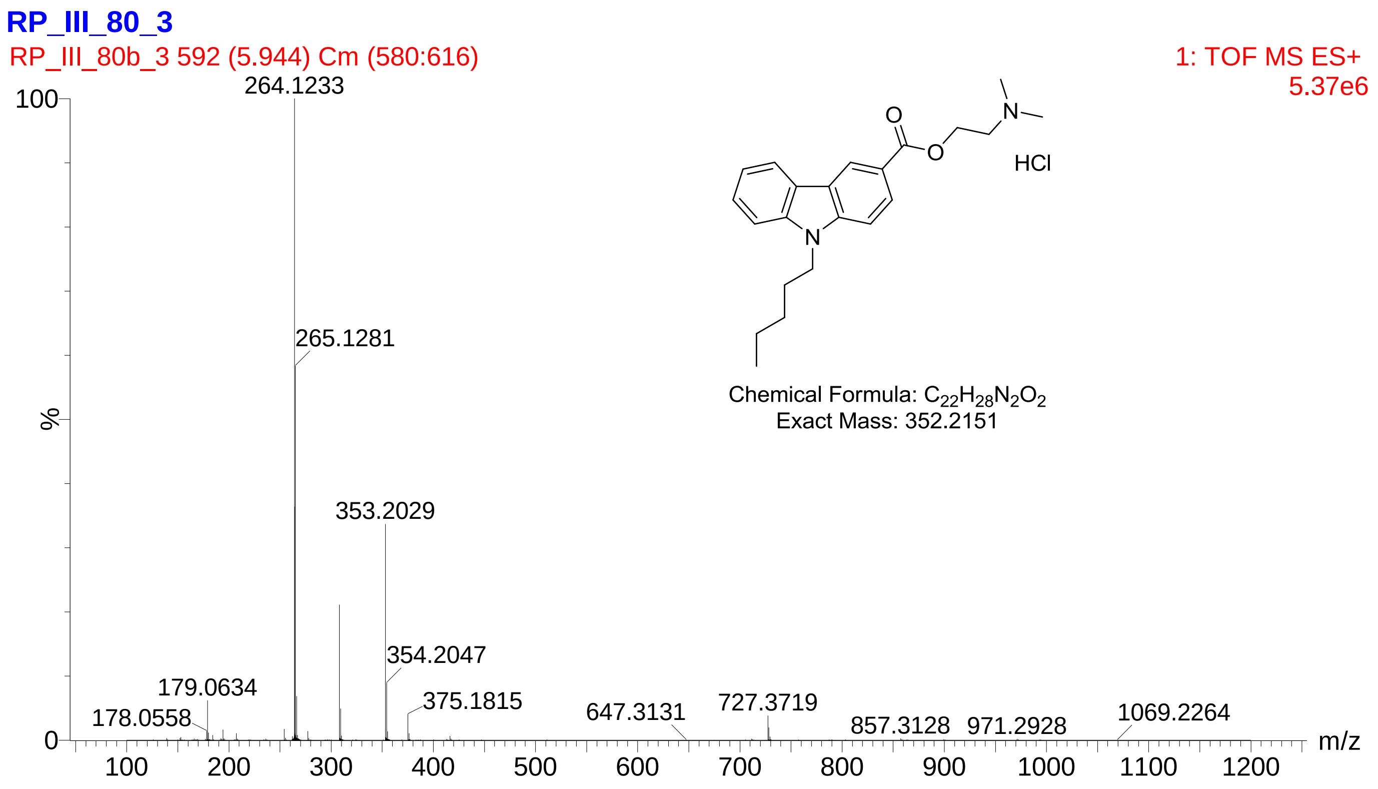

Supplement: Additional file 1 — Details on synthesis of compounds. [file 1744-8069-9-32-S1.pdf]
